# Graphene- and Carbon-Nanotube-Based Transparent Electrodes for Semitransparent Solar Cells

**DOI:** 10.3390/ma11091503

**Published:** 2018-08-22

**Authors:** Kyu-Tae Lee, Dong Hyuk Park, Hyoung Won Baac, Seungyong Han

**Affiliations:** 1 Department of Physics, Inha University, Incheon 22212, Korea; ktlee@inha.ac.kr; 2 Department of Chemical Engineering, Inha University, Incheon 22212, Korea; 3 Department of Electrical and Computer Engineering, Sungkyunkwan University, Suwon 16419, Korea; 4 Department of Mechanical Engineering, Ajou University, San 5, Woncheon-Dong, Yeongtong-Gu, Suwon 16499, Korea

**Keywords:** solar cells, semitransparent, CNT, graphene, transparent electrodes

## Abstract

A substantial amount of attention has been paid to the development of transparent electrodes based on graphene and carbon nanotubes (CNTs), owing to their exceptional characteristics, such as mechanical and chemical stability, high carrier mobility, high optical transmittance, and high conductivity. This review highlights the latest works on semitransparent solar cells (SSCs) that exploit graphene- and CNT-based electrodes. Their prominent optoelectronic properties and various fabrication methods, which rely on laminated graphene/CNT, doped graphene/CNT, a hybrid graphene/metal grid, and a solution-processed graphene mesh, with applications in SSCs are described in detail. The current difficulties and prospects for future research are also discussed.

## 1. Introduction

Semitransparent solar cells (SSCs), which exhibit eye-catching appearances by creating neutral- and multi-colors, have recently gained significant interest for their potential use in a variety of applications, such as power-generating windows for buildings and vehicles, high-efficiency tandem solar cells, power-generating color filters for displays, and wearable electronics [1,2,3,4,5,6,7,8,9,10,11,12,13,14,15,16,17,18,19,20]. Although the power-conversion efficiencies (PCEs) of the SSCs are lower than those of their opaque counterparts owing to a relatively thin photoactive layer and reduced reflections from the top electrode, sunlight can be harvested from both sides, and the installation of SSCs can be extended to a wide range of areas, both of which can counteract the low PCE of the SSCs. Achieving neutral-colored semitransparency and simultaneously generating electricity from the solar cells can be enabled by decreasing the thickness of the photoactive semiconductor layers and employing transparent conductive films for both the top and bottom electrodes. For the transparent conductive films, a thin metallic layer that exploits aluminum (Al), silver (Ag), or gold (Au) capped or sandwiched by highly transparent dielectric media has been demonstrated. However, easy oxidation of Al causing electrically non-conductive performance, and the low stability of Ag leading to performance degradation, are the challenges still to be addressed. In addition, Au is too pricy and has its interband transition at 470 nm, which results in strong absorption, thus limiting its practical application as the electrode. To resolve the challenges of the thin metals as the top electrodes, various transparent conductive oxides, such as indium–tin oxide (ITO), indium–zinc oxide (IZO), and aluminum-doped zinc oxide (AZO), have been employed. Although such transparent conductive oxides present high transparency and high conductivity as compared to the thin metals, high temperature during a deposition and a post annealing step is required, which can damage the underlying photoactive layer. This could be addressed by using solution-processed metal nanowires. Additionally, morphological control of the photoactive layer to produce a window-like appearance has also been explored [5,6,7]. However, microscale dewetted islands to produce transparency could induce scattering, and poor surface coverage of the photoactive layer could result in degraded electrical performance. In addition to neutral-colors, visually appealing, multi-color-generating solar cells, which can be harmoniously integrated with building envelopes, automobile surfaces, and existing display systems, have been demonstrated in various types of solar cells by exploiting surface plasmon resonances (SPRs) in metallic nanostructures, Fabry–Pérot resonances in multilayer thin-film structures and dielectric mirrors, and via bandgap engineering of the active semiconductor material [8,9,10,11,12,13,14,15,16,17]. As reflective or transmissive colors can be produced by incorporating various nanoarchitectures in the solar cells and the nanostructures typically involve the metals, it is highly desired that the nanostructures can also function as the electrode of a solar cell for easy integration. However, the thickness of the metal in the nanostructures is generally ultrathin to minimize the absorption loss in the metals, which yields low electrical conductivity. Hence, there is a strong need for a novel electrode material to improve both the optical and electrical performance of SSCs.

An enormous amount of effort is being devoted to the development of transparent conductive films employing thin metallic films, conductive polymers, metal oxides, metal meshes, nanowire networks, carbon nanotubes (CNTs), and graphene [18,19,20,21,22,23,24,25,26,27,28,29,30,31,32,33,34,35,36,37,38,39,40,41,42,43,44,45,46,47,48,49,50,51,52,53,54]. In particular, CNTs and graphene have received increasing attention because of their excellent optoelectronic properties, such as their high optical transparency, low sheet resistance, and high mobility. Moreover, they exhibit mechanical flexibility, environmental stability, and a low cost, thus showing great promise for transparent electrodes. Owing to these fascinating characteristics, there has been an increase in the number of studies on fabrication processes, modification methods, and patterning techniques of CNT- and graphene-based transparent electrodes for diverse optoelectronic devices [55,56,57,58,59].

This article reviews recent advances in the fabrication of transparent electrodes based on graphene and CNTs, as well as their characteristic performances and application, particularly in SSCs. Diverse approaches, which involve laminated stacked multilayer graphene, highly doped single-layer graphene, a hybrid monolayer graphene/metal grid, a solution-processed graphene mesh network, a laminated freestanding multi-walled CNT network, and p-doped single-walled CNTs, are examined. This paper concludes with challenges and future perspectives.

## 2. Graphene Electrode

### 2.1. Laminated Multilayer Graphene

Electrode materials that are visibly transparent and yet electrically conductive are a key component of a wide variety of electronic and optoelectronic devices, including solar cells. Doped metal oxides are the most common materials, such as indium–tin oxide (ITO), which offers a high transparency and low sheet resistance. However, owing to its chemical instability, its ceramic nature resulting in brittleness, and the scarcity of indium leading to high cost, C-nanomaterial-based transparent electrodes have emerged as an attractive alternative to these conventional transparent electrodes. Numerous studies have been conducted on the development of graphene-based transparent electrodes owing to their high electrical conductivity, high transparency, chemical stability, flexibility, and mechanical robustness. In this section, we discuss a laminated multilayer graphene-based transparent electrode for SSCs.

**Figure 1:**
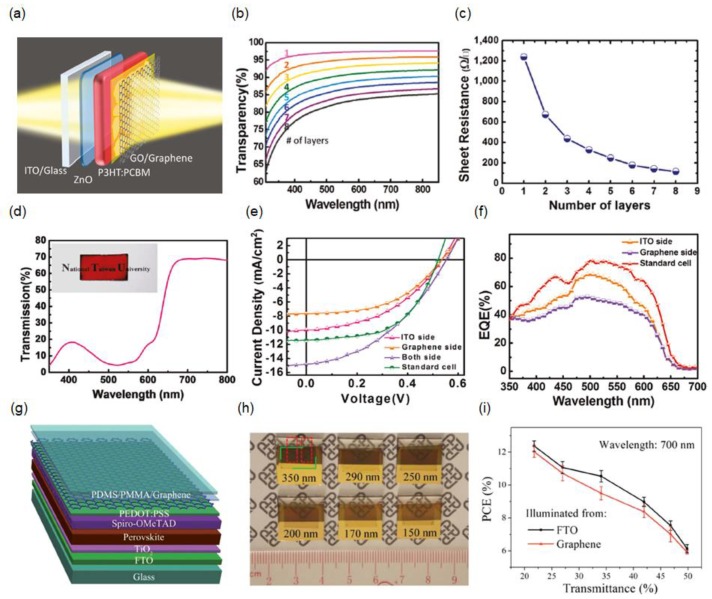
(**a**) Schematic view of the semitransparent inverted polymer solar cell with a structure of iridium–tin oxide (ITO)/ZnO/P3HT:PCBM/graphene oxide (GO)/graphene top electrode; (**b**) Transmission spectra and (**c**) sheet resistance for different numbers of graphene film layers on a glass substrate; (**d**) Transmission spectrum of the fabricated semitransparent polymer solar cell, along with a photograph of the device in the inset; (**e**) *J*–*V* characteristics and (**f**) external quantum efficiency (EQE) spectra of the standard cell and the semitransparent cell consisting of eight layers of graphene under light illumination from the ITO side and the graphene side. Reproduced with permission [64]. Copyright 2014, American Chemical Society; (**g**) Schematic illustration of the semitransparent perovskite solar cell with the transparent graphene top electrode; (**h**) Optical images of the semitransparent perovskite solar cells having transparent graphene electrodes with different thicknesses of the perovskite photoactive layers; (**i**) Average power-conversion efficiencies (PCEs) of the semitransparent perovskite solar cells with respect to the transmittance at 700 nm. Reproduced with permission [72]. Copyright 2015, Wiley.

Because it is difficult to achieve a sufficiently low sheet resistance with monolayer graphene, multilayer graphene films are typically needed to attain a low sheet resistance. Although the ITO’s bottom electrode is easily replaced by a graphene-based transparent electrode in organic and perovskite solar cells [60,61,62,63], it has been found to be critical to place the transparent top electrodes without damaging the underlying photoactive layer to create the SSC. Semitransparent inverted-type polymer solar cells, with the structure of ITO bottom electrode/zinc oxide (ZnO)/poly(3-hexylthiophene):phenyl-C_61_-butyric acid methyl ester (P3HT:PCBM)/graphene oxide (GO)/graphene top electrode, were demonstrated by employing a top-laminated chemical vapor deposition (CVD)-grown multilayer graphene electrode as shown in Figure 1a [64,65]. In this work, the graphene top electrode was thermally released during thermal annealing treatment of the photoactive layer, which can be used to manipulate the nanoscale morphology of the P3HT:PCBM blend film for improving the PCE of the polymer solar cells. Such a lamination process can be fully incorporated with the roll-to-roll (R2R) fabrication method for large-scale applications [66,67,68,69,70,71]. A CVD-grown graphene film is transferred by using a modified R2R process, where only one thermal release tape is used to obtain a four-layer graphene film, which can reduce the residual impurities between the graphene films. Figure 1b,c present measured transmission spectra at normal incidence and measured sheet resistance values of different numbers of graphene film layers, clearly showing that adding additional monolayer graphene reduces the transmission efficiency by approximately 2–3% and that the increasing the number of the graphene layers yields a low sheet resistance. Figure 1d displays the transmission spectrum of a fabricated semitransparent polymer solar cell, which exhibits a high transmission efficiency over wavelengths beyond 700 nm, where the photoactive layer is transparent. Its optical image exhibits a reddish color. Figure 1e presents the current–voltage curves and the corresponding external quantum efficiency (EQE) spectra of the standard cell that is completed with an optically thick Ag electrode and an SSC with the top electrode comprising eight layers of graphene under light illumination from the ITO side or the graphene side. Nearly 76% of the PCE of the standard cell, which is approximately 2.5%, is achieved by the semitransparent polymer solar cell with bifacial functionalities.

Owing to the many appealing characteristics of the graphene electrode described above, a laminated stacked multilayer graphene top electrode has been used to create semitransparent perovskite solar cells as shown in Figure 1g [72]. The SSC device’s structure consists of an electron-transporting TiO_2_ layer, a perovskite semiconductor, a hole-transporting 2,2′,7,7′-tetrakis(*N*,*N*-di-*p*-methoxyphenylamine)-9,9-spirobifluorene (Spiro-OMeTAD) layer, poly(3,4-ethylenedioxythiophene):poly(styrenesulfonic acid) (PEDOT:PSS), and a graphene top electrode. A thin layer of PEDOT:PSS film whose Fermi level (5.0 eV) is higher than the Dirac point of graphene (4.6 eV) is introduced, where more holes are injected into the graphene film by electrostatic doping, leading to an improved conductivity and hence a low sheet resistance of the graphene film [73,74,75,76]. Moreover, the adhesion between the graphene electrode and the perovskite photoactive layer can be improved by the PEDOT:PSS during the lamination process. Furthermore, the effect of the thin PEDOT:PSS layer on the transparency of the graphene electrode is trivial, as PEDOT:PSS is nearly transparent over the entire visible spectrum. Figure 1h presents optical images of the semitransparent perovskite solar cells with the two-layer CVD graphene top electrode having a photoactive layer thickness varying from 150 to 350 nm. The relationship between the transmittance at 700 nm and the PCE of the SSCs with the graphene electrode is presented in Figure 1i, indicating that approximately 50% of the transmittance at 700 nm and approximately 6% of the PCE are achieved by the SSC device with a 150-nm-thick photoactive layer. The demonstrated device performances surpass those of the SSCs in previous works, which is ascribed to the high optical transparency and high electrical conductivity of the graphene electrode [5,18,43,77,78,79,80,81,82,83,84].

### 2.2. Highly Doped Single-Layer Graphene

**Figure 2:**
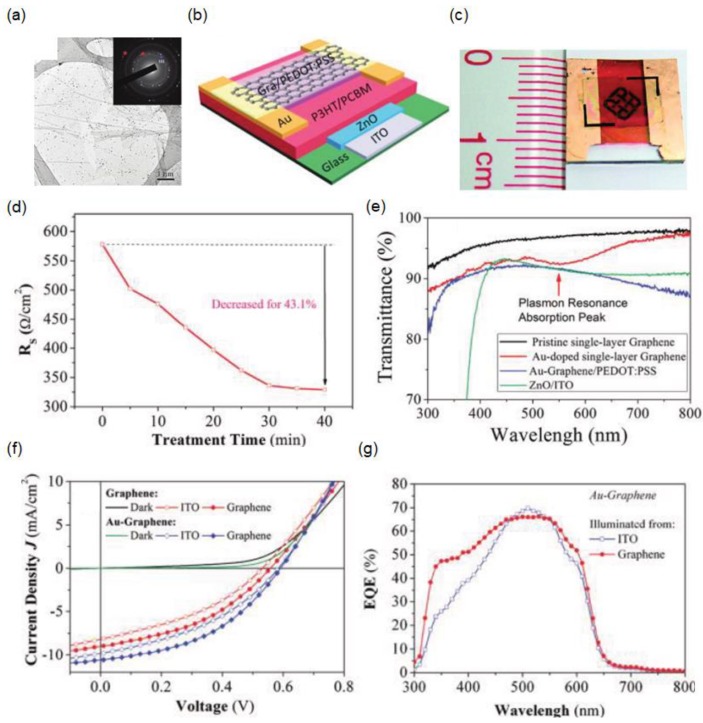
(**a**) TEM image of a single-layer graphene film doped with Au nanoparticles (NPs). The inset shows the diffraction pattern of the Au-doped graphene film, where red and blue circles correspond to the diffraction patterns of graphene and Au, respectively; (**b**) Schematic of the semitransparent organic solar cell consisting of glass/ITO/ZnO/P3HT:PCBM/PEDOT:PSS/graphene; (**c**) Photograph of the fabricated semitransparent solar cell (SSC) with the transparent graphene top electrode; (**d**) Sheet resistance of the single-layer graphene with respect to the treatment time in an HAuCl_4_ solution; (**e**) Light transmittance of single-layer graphene before and after Au doping, Au-doped graphene/PEDOT:PSS, and ZnO/ITO films on glass substrates; (**f**) *J*–*V* characteristics measured on both sides of the semitransparent organic solar cells with a pristine or Au-doped graphene top electrode under a solar simulator. The dark currents of the devices are presented as well; (**g**) EQE profiles measured on both sides of the semitransparent organic solar cell with an Au-doped graphene/PEDOT:PSS top electrode. The open and solid symbols correspond to the results for the ITO and graphene sides, respectively. Reproduced with permission [73]. Copyright 2011, American Chemical Society.

Although a single-layer graphene electrode can provide the highest transmission efficiency, its electrical conductivity is too poor to be used as an electrode. As described in the previous section, stacked multilayer graphene films can address the low conductivity. However, minimizing the residual resists and contaminants remains challenging during the graphene film transfer and stack processes. Another approach to achieving both a high transparency and high conductivity of the single-layer graphene film is to exploit effective chemical doping [73,75,85,86]. One example is graphene films immersed in an HAuCl_4_ solution, where the metal ions spontaneously decrease, resulting in the formation of Au nanoparticles (NPs) on the surface of the graphene films so that the surface potential of the single-layer graphene film can be tuned by controlling the treatment time [75]. In this case, the graphene film is p-doped, as it has been found that the surface potential of Au is higher than that of graphene. Figure 2a shows a transmission electron microscopy (TEM) image of the p-doped single-layer graphene film after chemical doping treatment with HAuCl_4_. The inset presents the diffraction pattern of the graphene film, which is found to be polycrystalline. Figure 2b presents a schematic of semitransparent organic solar cells with a p-doped single-layer graphene top electrode comprising glass/ITO/ZnO/P3HT:PCBM/PEDOT:PSS/graphene, and Figure 2c shows a photograph of a fabricated SSC having an active area of 5 mm × 4 mm for a solar-cell device. The device can be fabricated over such a large area because single-layer graphene is simple to prepare compared with laminated multilayer graphene films [50,61,64]. Figure 2d shows the relationship between the sheet resistance of the single-layer graphene film and the chemical doping treatment time, indicating that treatment for more than 30 min leads to a reduction of approximately 43% in the sheet resistance. A thin PEDOT:PSS layer is coated on the p-doped graphene film to increase the electrical conductivity of the graphene top electrode. By introducing Au NPs on the surface of graphene, the absorption arising from the SPR is observed at 540 nm, reducing the transmittance slightly, as shown in Figure 2e. Despite the reduction of the transmittance by the SPR, the transparency in the visible range of the p-doped single-layer graphene film with PEDOT:PSS is still higher (92%) than that of the multilayer graphene electrodes. Figure 2f,g illustrate the current density (*J*)–voltage (*V*) characteristics and the EQE spectra of the SSC with the graphene electrode and the Au and PEDOT:PSS co-doped graphene electrode under light illumination from both sides. The PEDOT:PSS doping leads to an enhanced fill factor (FF), which is attributed to the reduced sheet resistance of the graphene electrode, and also yields an improved open circuit voltage (*V_oc_*) due to the work-function increase caused by the p-type doping of the graphene electrode. PCEs of 2.7% and 2.2% are obtained from the SSC under illumination from the graphene side and the ITO side, respectively.

### 2.3. Hybrid Monolayer Graphene/Metal Grid

**Figure 3:**
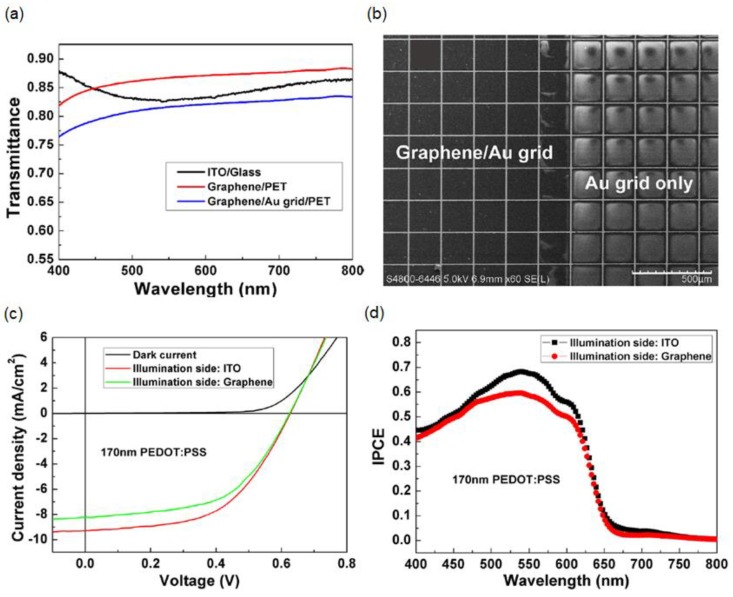
(**a**) Transmission spectra of three different electrodes: ITO/glass, monolayer graphene/PET, and monolayer graphene/Au grid/PET; (**b**) Top-view SEM image of the monolayer graphene/Au grid hybrid transparent electrode, where the size of the Au grid is 200 μm × 200 μm; (**c**) *J*–*V* characteristics and (**d**) incident-photon-to-electron conversion efficiency (IPCE) spectra of the semitransparent organic solar-cell devices measured under illumination from both sides of the semitransparent devices with a PEDOT:PSS thickness of 170 nm. Reproduced with permission [87]. Copyright 2013, American Physical Society.

Although SSC devices with a highly doped single-layer graphene electrode can be fabricated over a large area, the conductivity of the single-layer graphene electrode is limited. Additionally, ensuring the stability of chemically doped graphene electrodes remains a challenge. To overcome the aforementioned issues of the graphene electrode, a hybrid transparent monolayer graphene/metal grid was demonstrated as a top electrode for SSCs, exhibiting a significantly low sheet resistance and very good stability [87]. The semitransparent organic solar-cell device structure consists of glass/ITO/TiO_2_/P3HT:PCBM/PEDOT:PSS/single-layer graphene/Au grid/Polyethylene terephthalate (PET), where two separate devices (P3HT:PCBM/TiO_2_/ITO/glass and PEDOT:PSS/single-layer graphene/Au grid/PET) are prepared and then laminated. Photolithography with the lift-off technique is used to fabricate the Au grid with different grid dimensions ranging from 50 μm × 50 μm to 300 μm × 300 μm, with a grid line 10-μm wide on a flexible substrate, followed by a traditional solution method to transfer the CVD-grown single-layer graphene film to the top of the Au grid [73,88]. Then, part of the device is completed by coating the top of the single-layer graphene/Au grid with PEDOT:PSS doped with 50 mg/mL D-sorbitol. Finally, the two parts of the device are laminated together and thermally annealed at 120 °C with proper pressure. Both the sheet resistance and the optical transmittance of the single-layer graphene/Au grid electrode depend on the Au grid size, and a sheet resistance of 7–30 Ω/sq and a transmission efficiency of 66–84% at 550 nm are achieved. With Au grid dimensions of 200 μm × 200 μm, a transmission efficiency of 81.4% and a sheet resistance of 22±3 Ω/sq at 550 nm are obtained, both of which are comparable to those of the ITO electrode as presented in Figure 3a. Figure 3b shows a scanning electron microscopy (SEM) image of the fabricated Au grid hybrid electrode with (left) and without (right) single-layer graphene. The hybrid transparent single-layer graphene/Au grid electrode features a considerably improved conductivity, high stability, and mechanical flexibility compared with the monolayer graphene electrode. The *J*–*V* characteristics and incident-photon-to-electron conversion efficiency (IPCE) spectra of the SSC with the hybrid monolayer graphene/Au grid electrode under illumination from both sides are presented in Figure 3c,d, respectively. The FF initially increases with the increase of the PEDOT:PSS thickness, but the FF is reduced when the PEDOT:PSS thickness increases beyond 250 nm, which is attributed to the rough surface of the PEDOT:PSS causing bad contact of the interface between the photoactive layer and PEDOT:PSS. The optimized thickness of PEDOT:PSS was found to be 170 nm, which leads to the lowest series resistance and highest shunt resistance, yielding PCEs of 3.1% and 2.8% from the ITO side and the graphene side, respectively. The described hybrid transparent electrode can be employed for both the anode and cathode and can be integrated with the R2R process for large-area device fabrication.

### 2.4. Solution-Processed Graphene Mesh

**Figure 4:**
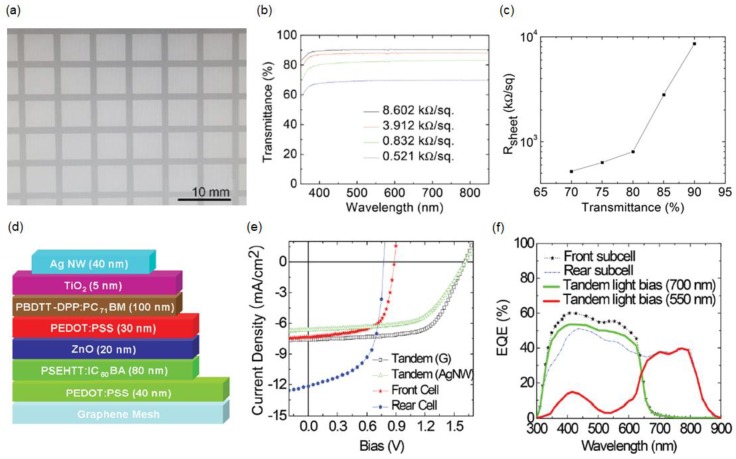
(**a**) Top-view SEM image of the graphene mesh; (**b**) Transmission spectra of the graphene mesh with the different sheet resistances; (**c**) Sheet resistance of the graphene mesh with respect to the light transmittance at 550 nm for various graphene mesh thicknesses; (**d**) Schematic of the semitransparent tandem cell; (**e**) *J*–*V* characteristics of front, rear, and tandem cells (light projected from the graphene mesh or Ag nanowires (NWs)); (**f**) EQE profiles measured under relevant bias illumination conditions. Reproduced with permission [90]. Copyright 2014, Wiley.

The conductivity of the single-layer graphene film can be significantly enhanced by incorporating the metal grid as discussed above. However, Au is not a good candidate for the transparent electrode, because the price of Au is high and vacuum deposition techniques must be used, limiting its potential for low-cost and large-area applications. Although other metals can be used to reduce the cost for practical applications, tuning the work function of the hybrid transparent monolayer graphene/metal grid electrode can be an issue. In addition, Au shows strong absorption of light at 470 nm owing to the presence of the interband transition as well as a low transmission efficiency in the near-infrared (NIR) wavelength region [89]. Furthermore, the high reflection of the metal grid generally results in low transparency with non-negligible scattering, which is not desirable for SSC and tandem solar-cell applications. To address these challenges, a solution-processed graphene mesh electrode for semitransparent tandem solar cells was demonstrated [90]. To achieve a sufficiently high electrical conductivity of the graphene mesh electrode, multilayer GO was spin-casted and then patterned using a standard photolithography technique [91]. Figure 4a presents a top-view SEM image of the transparent graphene mesh conducting electrode. Figure 4b,c show the transmission spectra of the graphene mesh with different sheet resistances and the sheet resistance of the graphene mesh versus the transmittance at 550 nm, respectively, revealing that a transmittance of 90% (70%) is achieved with a sheet resistance of 8.6 (0.52) kΩ sq^−1^. Figure 4d presents a schematic representation of the semitransparent tandem solar cells, where a bulk heterojunction front subcell comprises graphene mesh/PEDOT:PSS/poly[(4,4′-bis(3-ethyl-hexyl)dithieno[3,2-b:″3′-d]silole)-2,6diyl-alt-(2,5-(3-(2-ethyl-hexyl)thiophen-2-yl)thiazolo[5,4-d]thiazole] (PSEHTT) (electron donor)/indene-C_60_-bisadduct (electron acceptor)/ZnO/PEDOT:PSS (interconnecting layer), and a rear subcell consists of poly {2,6′4,8-di(5-ethylhexylthienyl)benzo[1–b;3,4-b]dithiophene-alt5-dibutyloctyl-3,6-bis(5-bromothiophen-2-yl)pyrrolo[3–c] pyrrole-1,4-dione} (PBDTT-DPP)(electron donor)/[6,6]-phenyl-C_71_-butyric acid methyl ester (PC_71_BM) (electron acceptor)/TiO_2_/Ag nanowire electrode [92,93,94]. Integrating the two semiconductors with different bandgaps (1.80 and 1.44 eV) allows the absorption to be broad, up to 900 nm, allowing the semitransparent tandem solar cell to achieve a PCE of 8.02%. Figure 4e demonstrates the *J*–*V* characteristics of a single-junction front subcell exhibiting a PCE of 4.29% with an 80-nm-thick PSEHTT:IC_60_BA layer, a rear subcell exhibiting a PCE of 5.60% with a 100-nm-thick PBDTT-DPP:PC_71_BM layer, and tandem solar cell exhibiting a PCE of 8.02%. Figure 4f displays the EQE spectra of the front subcell, rear subcell, and tandem solar cell, where the tandem solar cell is measured with two optical-bias light sources at 550 and 700 nm. Both subcells generate matched photocurrents, which are desired for the tandem solar cells, and the EQE spectra agree well with the absorption spectra of the front and rear subcells. A semitransparent tandem solar cell with improved transparency (44.90%) compared with previous works is achieved, without affecting the PCE [95,96,97].

### 2.5. All-Graphene Electrodes

**Figure 5:**
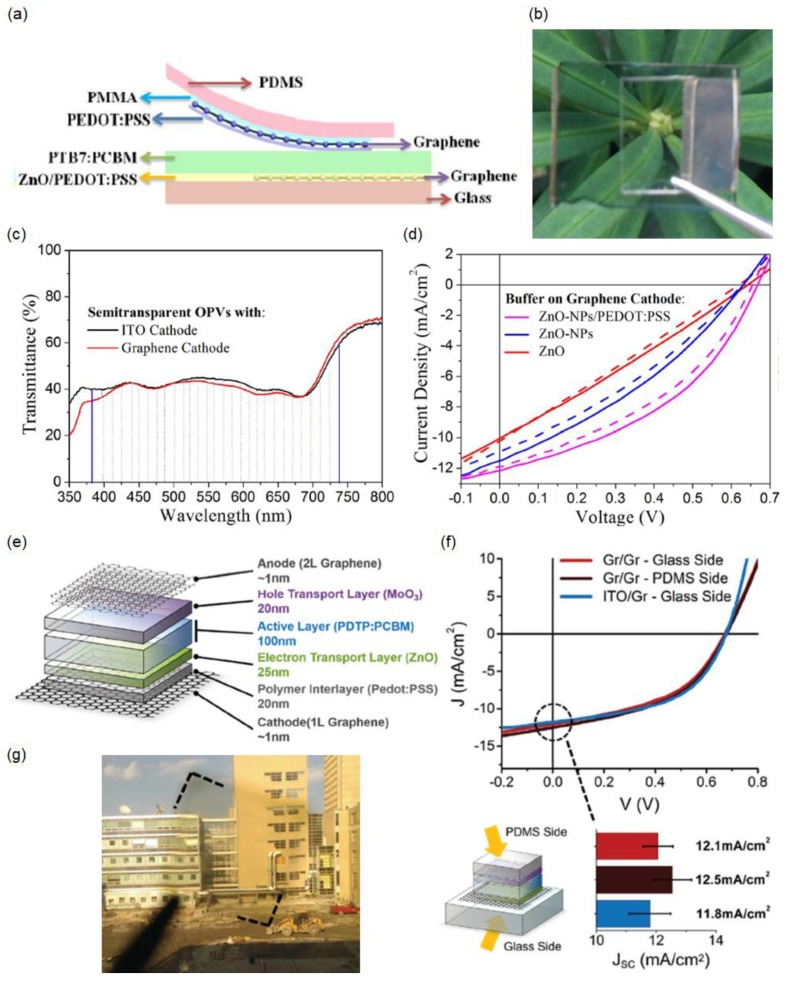
(**a**) Schematic illustration of the semitransparent organic solar cell with all-graphene electrodes; (**b**) Neutral-colored SSC with all-graphene electrodes; (**c**) Transmission profiles of two semitransparent organic solar cells with graphene anodes and graphene or ITO cathodes. The dashed lines indicate the visible region; (**d**) *J*–*V* characteristics of the SSC with all-graphene electrodes. The graphene cathodes were modified with different buffer layers. The devices were illuminated and characterized from the anode (solid line) and cathode (dash line). Reproduced with permission [98]. Copyright 2015, American Chemical Society; (**e**) Device structure with approximate layer thicknesses; (**f**) *J*–*V* curves of the PC_70_BM graphene/graphene devices illuminated from the glass (cathode) side versus the polydimethylsiloxane (PDMS) (anode) side. The *J*–*V* curve of a PC_70_BM ITO/graphene device illuminated from the glass/ITO side is included for reference. The lower-right bar chart shows the average *J_sc_* values for these configurations; (**g**) Photograph taken through a PC_60_BM device. The dotted lines outline the corners of the device. Reproduced with permission [99]. Copyright 2015, Wiley. PMMA: Poly(methyl methacrylate).

Recent studies have also demonstrated the fabrication of SSCs with graphene transparent electrodes as both the anode and cathode, which can potentially improve the optical transparency, mechanical flexibility, and stability of SSCs [98,99]. Additionally, C-based SSC devices can be inexpensive and eco-friendly. Figure 5a presents a schematic view of semitransparent organic solar cells with all-graphene electrodes comprising two-layer stacked graphene [98]. Although ZnO—a n-type oxide semiconductor—can lead to n-type doping of the graphene film, direct coating of ZnO on graphene is found to have a destructive effect and therefore yields a high sheet resistance. To protect the graphene film, a thin layer of PEDOT:PSS is coated on top of the graphene, followed by a thin layer coating of ZnO NPs that are synthesized via the hydrothermal method, for preparation of the graphene cathode [100,101,102]. For the graphene anode, PEDOT:PSS with the optimal thickness (~50 nm) is coated on top of the graphene film after the graphene surface is treated by oxygen plasma for improved uniformity [103]. The graphene anode is conformally laminated on top of the organic photoactive layer, polythieno[3,4-b] thiophene/benzodithiophene (PTB7) and PC_71_BM [104]. A photograph of the fabricated SSC with all-graphene electrodes, apparently showing a neutral-colored background image through the device, is shown in Figure 5b. Figure 5c displays the transmission spectrum of the SSC device with the graphene cathode, which exhibits a similar transmission profile to the ITO cathode. Both devices present an average visible transmittance of ~40%, which allows diverse applications, such as power-generating windows and tandem solar cells. The high average optical transmittance in the visible region is attributed to the high transmission efficiency of the all-graphene electrodes and the thinness of the organic photoactive layer (~90 nm). The average visible transmittance can be improved by decreasing the thickness of the photoactive layer, sacrificing the PCEs of the SSC devices. Figure 5d presents the *J*–*V* characteristics of three SSC devices with different buffer layers on both sides of the graphene cathode. The best solar-cell performance, which is due to the highest electrical conductivity, is achieved with the graphene cathode that is modified with the ZnO NPs/PEDOT:PSS buffer layer, where a PCE of 3.35% (3.06%) is attained under light illumination from the top (bottom) graphene electrode.

Although the optical transparency of the SSC device can be improved by reducing the thickness of the organic photoactive layer, the PCEs are reduced, which is not desired. For enhancing the optical transmittance while preserving or even enhancing the PCEs, an SSC wherein the organic active layer mainly absorbs light in the ultraviolet (UV) and NIR wavelength ranges with a single (two)-layer graphene cathode (anode) was demonstrated [99]. In this study, the SSC device structure consists of single-layer graphene cathode/PEDOT:PSS/ZnO/poly[2,7-(5,5-bis-(3,7-dimethyloctyl)-5Hdithieno[3,2-b:20,30-d]pyran)-alt-4,7-(5,6-difluoro-2,1,3-benzothiadiazole)] (PDTP-DFBT):[6,6]-phenyl-C_71_-butyric acid methyl ester (PC_70_BM)/molybdenum trioxide (MoO_3_)/two-layer graphene anode. Because PDTP-DFBT mainly absorbs light in the wavelength range of 600 to 900 nm whereas PC_70_BM primarily absorbs the shorter wavelengths of visible light, high transparency in the visible wavelength region can be accomplished by a bulk heterojunction blend, as shown in Figure 5g, revealing little color difference across the fabricated SSC device [105,106]. An overall transmission efficiency of 59% at 550 nm and an average visible transmittance of 54% are achieved by the SSC device with PDTP-DFBT:PC_70_BM. An improved optical transmittance but a reduced PCE of the SSC device can be obtained by using PDTP-DFBT:PC_60_BM. Figure 5f shows the *J*–*V* curves of the two SSC devices with all-graphene electrodes and an ITO cathode and a graphene anode (the inset schematic shows the SSC device configuration). The highest short-circuit current density (*J_sc_*) of 12.5 mA cm^-2^, leading to a PCE of 4.1%, is attained from the SSC with all-graphene electrodes under illumination from the top plastic stamp side.

## 3. CNT Electrode

### 3.1. Laminated CNT

**Figure 6:**
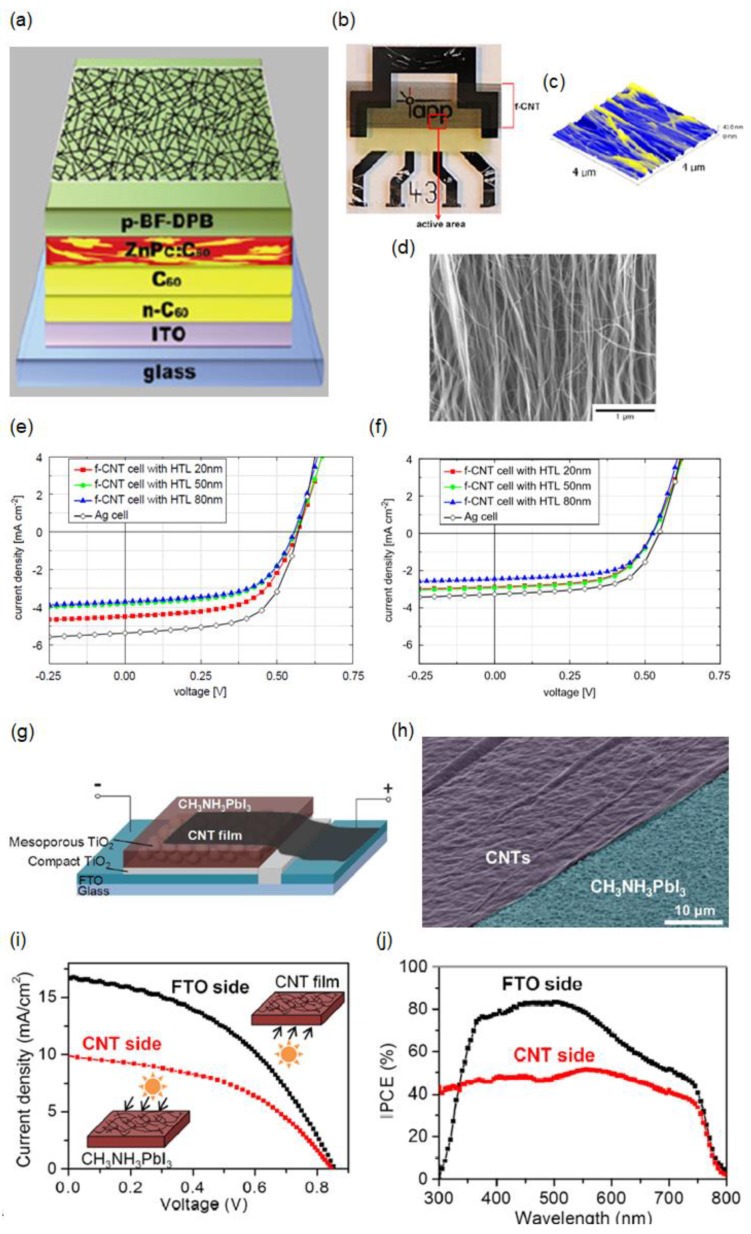
(**a**) Schematic of the SSC with the transparent freestanding multi-wall carbon nanotube (f-CNT) top electrode; (**b**) Photograph of the f-CNT cell; (**c**) AFM image (4 μm × 4 μm); (**d**) SEM image of the f-CNT sheet on top of the solar cell. *J*–*V* characteristics of the Ag reference cell and f-CNT cells with different hole-transporting layer (HTL) thicknesses (20, 50, and 80 nm) during (**e**) bottom and (**f**) top illumination. Reproduced with permission [44]. Copyright 2012, Elsevier; (**g**) Device structure of a CH_3_NH_3_PbI_3_ perovskite solar cell with the CNT film electrode; (**h**) Tilted SEM image of the CH_3_NH_3_PbI_3_ perovskite substrate (blue) partly covered by a CNT film (purple); (**i**) *J*–*V* curves of a CH_3_NH_3_PbI_3_ perovskite/CNTs solar cell with illumination from the fluorine-doped tin oxide (FTO) side and from the CNT side under conditions of AM1.5 100 mW cm^−2^; (**j**) IPCE spectra of a CH_3_NH_3_PbI_3_ perovskite/CNTs solar cell with illumination from the FTO and from the CNT side. Reproduced with permission [43]. Copyright 2015, American Chemical Society.

In addition to graphene-based transparent electrodes, there have been increasing efforts to develop CNT-based transparent electrodes, as CNT is chemically stable, mechanically flexible, electrically conductive, solution-processable, and transparent compared with the other transparent conductive oxides (TCOs). The lamination of a CNT network film as a top electrode for semitransparent small-molecule organic solar cells has been reported, where the fabrication of the transparent electrodes without damaging the underlying organic photoactive layer is crucial [44]. In this work, freestanding multi-wall CNT (f-CNT) sheets are prepared using the conventional CVD process, and then the CNT network film lamination is performed followed by a densification process to improve the transparency and conductivity [107]. Figure 6a depicts the device structure, which consists of glass/ITO cathode/electron transporting tetrakis(1,3,4,6,7,8-Hexahydro-2H-pyrimido[1,2-a]pyrimidinato)ditungsten (II) (W_2_(hpp)_4_) doped C_60_ layer/C_60_/zinc phthalocyanine (ZnPc):fullerene C_60_ bulk heterojunction photoactive layer/hole transporting 2,2′-(perfluoronaphthalene-2,6-diylidene)dimalononitrile (F_6_TCNNQ) doped *N*,*N*′-((diphenyl-*N*,*N*′-bis)9,9,-dimethyl-fluoren-2-yl)-benzidine (BF-DPB) layer/f-CNT. Optical, atomic force microscopy (AFM), and SEM images of the f-CNT film, which has a predominant orientation, laminated on top of the SSC after the densification process are presented in Figure 6b–d, respectively [108]. Figure 6e,f show the *J*–*V* characteristics of the semitransparent small-molecule organic solar cells with different thicknesses of the hole-transporting layer under light illumination from the bottom (ITO cathode) and top (f-CNT anode) sides, respectively. PCEs of 1.3% and 0.9%, along with an average visible transmittance of 27% with negligible scattering effects, are achieved by the SSCs with the 50-nm-thick hole-transporting layer under bottom and top illumination, respectively, both of which present comparable PCEs of the reference cell (1.9% and 1.0% for the bottom and the top illuminated cells, respectively), where a thin metal layer is used for the top electrode instead of f-CNT. One of the challenges of the CNT electrodes is that their surface is typically fairly rough, causing electrical shortage of the solar cells, which can be resolved via a planarization method with PEDOT:PSS [109,110]. In contrast, it is observed that all the devices with an f-CNT top electrode present a very low leakage current, which is attributed to the fact that additional and sizable shunt paths are not generated during the room-temperature orthogonal liquid-solution-assisted self-laminating process of the f-CNT top electrode. This implies that the deposition of the f-CNT film on top of the photoactive layer is uniform, without structural defects and charge-carrier trapping sites.

A laminated CNT network film as a top electrode was also demonstrated in semitransparent perovskite solar cells [43]. Figure 6g presents the perovskite solar-cell device structure, where a thin mesoporous nanocrystalline layer of TiO_2_ is used as a scaffold. A sequential deposition method is employed to deposit the perovskite photoactive layer, and a floating-catalyst CVD method is used to synthesize the thin f-CNT network film that is transferred on top of the light-absorbing perovskite semiconductor in an ambient atmosphere at room temperature [111,112]. A small drop of toluene allows the surface tension that occurs during the vaporization of toluene to pull the thin CNT network film towards the perovskite semiconductor via the van der Walls force without the dissolution of the photoactive layer, leading to conformal transfer of the CNT film and hence an improved electric contact. Figure 6h shows a tilted top SEM image of the thin f-CNT network film (purple) conformally coated on top of the light-absorbing perovskite semiconductor (bluish green). The *J*–*V* characteristics and the IPCE profiles of the semitransparent perovskite solar cells with the CNT top electrode under illumination from both sides are presented in Figure 6i,j, respectively. PCEs of 6.29% and 3.88% are achieved by the SSC under illumination from the glass and CNT sides, respectively, where the transferred CNT network film presents not only a lower transmission efficiency but also a lower charge-collection efficiency, yielding lower absorption in the photoactive layer and therefore a lower *J_sc_*. This is confirmed by the IPCE spectra presented in Figure 6j. By eliminating the structural defects of the CNT film and incorporating Spiro-OMeTAD for better coverage and charge collection, the PCE can be further improved.

### 3.2. Doped CNT

**Figure 7:**
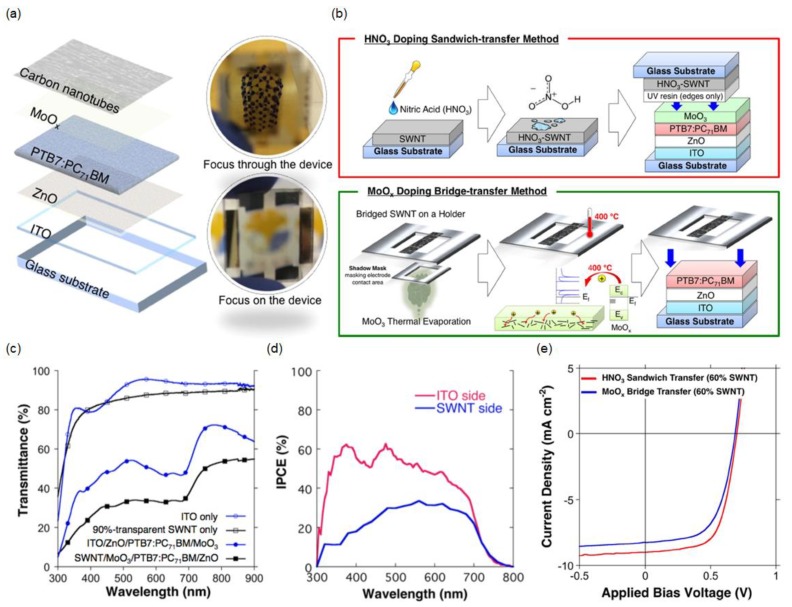
(**a**) Schematic diagram of the SSC with the laminated CNT top electrode (left) and photographs with different foci (right); (**b**) Schematic illustrations of the HNO_3_ doping sandwich transfer process (above) and the MoO_x_ thermal doping bridge transfer process (below); (**c**) Transmission spectra of glass/ITO, glass/90%-CNT, glass/ITO/ZnO/PTB7:PC_71_BM/MoO_3_, and glass/90%-CNT/MoO_3_/PTB7:PC_71_BM/ZnO; (**d**) IPCE measurement spectra of the SSC devices when light is shone from the ITO side and when light is shone from the CNT side; (**e**) *J*–*V* curves of 60% transparent CNT-based SSC devices fabricated using the HNO_3_ sandwich transfer method (red line) and the MoO_x_ bridge transfer method (blue line). Reproduced with permission [113]. Copyright 2016, Nature Publishing Group. SWNT: Single-walled carbon nanotube.

Although the semitransparent performance can be accomplished by using the laminated CNT film as the top electrode, the lamination process is not easy, and the sheet resistance of the laminated CNT film is high for practical applications. Recent work revealed neutral-colored semitransparent organic solar cells, where the CNT films were p-doped with HNO_3_ via the sandwich transfer method and MoO_3_ via the bridge transfer process [113]. Figure 7a shows a schematic illustration and optical images of the window-like semitransparent organic solar cells comprising glass/ITO/ZnO/PTB7:PC_71_BM/MoO_x_/CNT, where the CNT network films with high purity and long nanotube bundle lengths were prepared via the aerosol CVD method, and the doping was performed on the CNT films through the direct lamination and dry lamination methods [114,115]. Although HNO_3_ acid is an effective p-type dopant, direct application is not possible owing to the high acidity of HNO_3_ [116]. The HNO_3_ doping sandwich transfer procedure is illustrated in Figure 7b (top). A drop of HNO_3_ is placed on the CNT film followed by post-annealing treatment at 80 °C for 5 min, which causes the CNT film to appear reddish, indicating that the CNT network film is p-doped. This can be validated by checking the reduction in the Fermi level value from −5.0 to −6.0 eV through photoelectron yield spectroscopy (PYS). Then, the HNO_3_-doped CNT network film is placed onto a MoO_3_ film, where UV resin is used to promote the adhesion between the HNO_3_–CNT film and MoO_3_. When the device is illuminated from the ITO side, a PCE of 3.7% is achieved. Figure 7b (bottom) presents the MoO_x_ doping bridge transfer process, which is found to be more stable than the HNO_3_ sandwich transfer method [40]. The CNT film is bridged on a metal holder, as shown in the figure, and MoO_3_ is thermally evaporated with a shadow mask to pattern the contact area of the electrode followed by post-annealing treatment at 400 °C. The p-doping of the CNT network film is confirmed by measuring the Fermi-level value, which decreases from −5.0 to −5.6 eV, using PYS. The MoO_x_-doped CNT film is transferred directly onto the photoactive layer of the partially fabricated organic solar-cell device, achieving a PCE of 3.1% under light illumination from the ITO side. The transmission spectra of glass/ITO, glass/pristine CNT film exhibiting an average visible transmittance of ~85%, glass/ITO/ZnO/PTB7:PC_71_BM/MoO_3_, and glass/CNT/MoO_3_/PTB7:PC_71_BM/ZnO exhibiting an average visible transmittance of ~40% are presented in Figure 7c. Figure 7d shows the IPCE spectra of an SSC with the p-doped CNT electrode under illumination from the ITO and CNT sides. Although the pristine CNT network film shows a high transparency of ~90%, relatively low PCEs are achieved, and the fabricated devices exhibit unstable *J*–*V* characteristics that are attributed to the mechanical variability and the sensitivity of the transfer methods for the CNT film doping. Such instability of the electrical properties with the thin CNT film can be easily resolved by increasing the thickness of the original CNT network film, which can also improve the electrical conductivity despite the reduced average visible transmittance. By using thicker CNT films that show a transmission efficiency of ~60% at 550 nm, stable *J*–*V* curves are obtained, with PCEs of 4.1% and 3.4% for SSC devices with HNO_3_-doped and MoO_x_-doped CNT electrodes, respectively. The thicker CNT network film is less sensitive to the microwrinkles formed during the transfer process, leading to a higher *V_oc_*. In addition to the improved *V_oc_*, the enhanced electrical conductivity of the thicker CNT film yields a higher FF. Improved PCEs can be attained by sacrificing the transparency of the device.

## 4. Conclusions

The PCEs of SSC devices are significantly lower than those of the conventional solar cells with an opaque electrode. For the further efficiency enhancement of the SSC, various schemes can be exploited: (1) increased light absorption due to light-trapping effects in plasmonic nanostructures and nanostructure-textured surfaces; (2) broadband anti-reflection coatings yielding increased absorption in the photoactive layer and thus increased photocurrent generation; (3) integration of perfect light-absorbing mechanisms in the infrared (IR) regime for thermophotovoltaic energy-conversion applications; and (4) the design of tandem solar cells wherein more semiconductors, which can efficiently harvest the UV and IR portions of the solar spectrum rather than visible light, are employed. In addition to an SSC with a window-like appearance, multi-colored SSC devices can be developed for aesthetic solar panels and power-generating color filters for display technologies. Further enhancing the PCEs of SSCs and incorporating more functionalities into SSCs can introduce new avenues for the commercialization of SSCs.

Regarding the transparent electrodes that have been widely used in a variety of the optoelectronic devices, the TCOs—including ITO—are still dominant in the market, but their brittleness and expensive fabrication remain a challenge, restricting their potential in many future flexible and wearable applications. Although considerable effort is directed towards the development of transparent electrodes based on conducting polymers, their poor stability and durability are significant issues. Owing to their high transparency with optically neutral colors, high electrical conductivity, and excellent mechanical flexibility, both CNT and graphene—allotropes of C—have been extensively explored for transparent electrode materials. Various fabrication methods, modification approaches, and patterning strategies for CNT- and graphene-based transparent electrodes for diverse electronic and optoelectronic devices have been demonstrated. However, there are many challenges to be overcome for the industrialization of both CNT- and graphene-based transparent electrodes for numerous optoelectronic applications. First, both the optical transparency and electrical conductivity of the CNT- and graphene-based transparent electrodes must be improved, both of which are still lower than those of the ITO electrode. This is very important for SSC applications. New fabrication methods for preparing CNT and graphene with high quality, different morphologies, and a rapid growth rate should be developed. The second challenge is creating CNT- and graphene-based transparent electrodes over a large area with reduced costs, which is essential for commercialization. Third, although significant efforts have been devoted to investigating various dopants for enhancing the conductivity of CNT and graphene, these dopants have poor stability, which should be resolved, especially for SSC applications that require long-term stability. Furthermore, creating a smooth surface of the CNT- and graphene-based transparent electrodes remains a bottleneck; it is difficult to achieve clear background images through the SSC devices, limiting their widespread use in practical applications. To industrialize the CNT- and graphene-based transparent electrodes for SSC applications, the aforementioned challenges should be addressed. Despite these issues, we envision a bright future for CNT- and graphene-based transparent electrodes for SSC applications, owing to their unique characteristics that are unattainable with other transparent electrode approaches.
